# Enhanced Intensity Dependence as a Marker of Low Serotonergic Neurotransmission in High Optimistic College Students

**DOI:** 10.1155/2013/793673

**Published:** 2013-12-08

**Authors:** Jibiao Zhang, Daxing Wu, Shuqiao Yao, Yunxuan Xu, Xuejing Lu

**Affiliations:** ^1^Medical Psychological Institute, The Second Xiangya Hospital of Central South University, No. 139, Middle Renmin Road, Changsha, Hunan 410011, China; ^2^Department of Psychology, Macquarie University, Sydney, NSW 2109, Australia

## Abstract

Positive psychology focuses were on the merits of individuals, such as optimism and positive attitude, and the subsequent cultivation of these virtues. Optimism or pessimism is a significant predictor of physical health outcomes. The present study examined whether optimism or pessimism is associated with the loudness dependence of auditory evoked potentials (LDAEP), a biological indicator of serotonergic neurotransmission, for the N1, P2, and N1/P2 peaks in college students. The amplitudes and amplitude-stimulus intensity function (ASF) slopes of the N1, P2, and N1/P2 peaks were determined in the 24 (10 males) high optimistic and 24 (14 males) high pessimistic individuals. Significantly higher P2 ASF slopes were found in the optimistic group relative to the pessimistic group. Concerning peaks and ASF slopes of N1 and N1/P2, no significant differences were observed. Our results suggest that the serotonergic neurotransmission of the high optimistic college students was inferior to that of the pessimistic ones. Further investigations are needed to provide sufficient support for our results.

## 1. Background

The field of positive psychology has become increasingly popular over the past several decades. Positive psychology focuses were on the merits of individuals, such as optimism and positive attitude, and the subsequent cultivation of these virtues. Optimists are individuals who expect the occurrence of favorable events, despite negative situations, and maintain a positive mood and attitude. Conversely, pessimists are those who expect inauspicious events and hold on to negative feelings such as anxiety, anger, and sadness [1]. Research on the relationship between personality characteristics and physical health has examined these viewpoints. Optimistic individuals often take a positive or optimistic attitude in life, while the pessimistic individuals are more inclined to hold a negative or pessimistic coping style. In daily life, people often overestimate the likelihood of positive events but underestimate that of negative events that happen to themselves by only updating beliefs of positive information rather than negative information [2]. In a meta-analysis, Rasmussen et al. reported that optimism is a significant predictor of positive physical health outcomes [3]. Optimism or pessimism can predict depressive symptoms and decrease the cumulative incidence of depressive symptoms [4–6].

Loudness or intensity dependence of auditory evoked potentials (LDAEP/IDAEP) has been shown to serve as a biological indicator of serotonergic neurotransmission [7]. This electroencephalographic trait describes the changes of N1 and P2 amplitudes of auditory evoked potentials when tones of different intensities are presented. Generally, the ratio of the amplitudes when different stimulus intensities arise is stable for the listener, and can be seen in the amplitude of the N1/P2 components. This finding is observed less frequently in the N1 or P2 peaks as well [8]. The slope of the amplitudes is thought to be inversely related to serotonergic activity. The first convincing evidence for this hypothesis came from an animal study conducted by Juckel et al. (1997), in which N1/P2 amplitudes were reported to be mediated by serotonin (5-hydroxytryptamine, 5-HT) receptors in the primary auditory cortex [9]. Several studies have demonstrated that serotonergic medications can influence the LDAEP of healthy human subjects [10, 11].

Optimism is primarily correlated to extraversion and positive emotions [12–14]. There have been some studies which have correlated extraversion (or related constructs, such as novelty seeking, hypomanic personality (HYP), and hyperthymic temperament (HYT)) with LDAEP. A study by Friedman and Meares (1979) found a positive correlation of extraversion with intensity dependence of visual stimuli [15]. Similarly, novelty seeking correlated positively with LDAEP of the tangential dipole of the auditory cortex in healthy subjects in a study by Juckel et al. (1995) [16]. Recently, Hensch et al. reported that both HYP and HYT were significantly correlated with a steeper LDAEP [17]. Besides, Fox et al. reported that individuals with the homozygous long allele (LL) genotype of the 5-hydroxytryptamine transporter (5-HTT) gene showed a marked bias when selectively processing positive affective material alongside selective avoidance of negative affective material, whereas such potentially protective activity was absent among individuals carrying the short allele (SS or SL) [18]. Strobel et al. and Hensch et al. observed a steeper LDAEP in LL subjects [19, 20].

Therefore, we speculate that optimistic individuals, similar to the LL individuals, may display a stronger LDAEP than pessimistic individuals. The aim of this study was to investigate whether two different personality traits (being high optimistic/pessimistic) are associated with different LDAEP in healthy college students.

## 2. Materials and Methods

### 2.1. Behavioral Measures

The Chinese Version of the Optimism-Pessimism Scale (OPS-C), the Self-rating Depression Scale (SDS), and the Self-rating Anxiety Scale (SAS) were administrated to all the participants.

The Optimism-Pessimism Scale (OPS), originally developed by Dember et al. [21], was designed to measure optimistic and pessimistic traits. Xu et al. later introduced a culture specific version into China [22]. Each item is rated on a 4-point scale (1 = most agree; 4 = most disagree). The positive items were scaled inversely and the total scores of the scale were the sum of positive and negative items. The higher the scores are the more optimistic a person is and vice versa. The Cronbach's alpha coefficients of the total scores, the optimism subscale and the pessimism subscale, were 0.819, 0.791, and 0.751, respectively. The correlation coefficient between the total scale and the optimism subscale was 0.825, while the correlation between the total scale and the pessimism subscale was −0.803.

The SDS [23] and the SAS [24] consist of 20 items each. Each item is rated on a 4-point scale (1 = none or scarcely; 4 = most or all the time). The higher the total scores are, the greater the levels of depression or anxiety are. Scores of SDS and SAS were correlated to the OPS-C in our previous research [22], so, the SDS and the SAS were administrated to all the subjects.

### 2.2. Procedure

A total of 503 college age students from Changsha College, China, completed study questionnaires, containing the OPS-C, SDS, and SAS. For the purposes of this study, participants whose total OPS-C scores fell into the top 16% (above 100) were defined as high optimistic, while those whose total scores fell in the bottom 16% (below 90) were defined as high pessimistic. 25 of those in the top 16% and 25 of those in the bottom 16% with normal hearing were contacted and agreed to participate in the study. A self-compiled interview was conducted to ensure that participants did not have a history of neurologic or psychiatric disorders, or drug or alcohol abuse. All participants gave written informed consent. The research protocol was approved by the Second Xiangya Hospital Ethics Committee. One male in the optimistic group was rejected for the reason that the impedance could not be reduced below 5 kΩ, and one male in the pessimistic group was dropped out due to sleeping during the experiment. So, a total of 24 (10 males) high optimistic and 24 (14 males) high pessimistic individuals were included in the final analysis.  Table 1 displays the participants' characteristics.

### 2.3. ERP Recordings

EEG data were recorded in a sound-attenuated and electrically shielded room. Subjects were seated in a comfortable armchair and were asked to look at the wall 2 meters in front of them. Auditory stimuli of 1000 Hz and 40 ms duration (10 ms rise/fall) were presented at 60, 70, 80, 90, and 100 dB/SPL via headphones (Telephonic Inc.) in a pseudorandomized order. Neuroscan Stim 2.0 software generated stimulus presentation. Evoked potentials were recorded from 26 scalp electrodes according to the international 10/20 systems (impedance below 5 kΩ) with linked ear-mastoid as a reference using a 32-channel EEG amplifier (SynAmps, 32 EEG/EP, Neuroscan Inc., El Paso, TX, USA). Electrodes placed above and below the left eye and on the right and left outer canthi were used to detect eye artifacts. EEG data were recorded with a sampling rate of 500 Hz in the frequency range of 0.1 to 100 Hz.

### 2.4. EEG Data Analysis

Offline EEG were filtered with a 0.1 to 30 Hz (24 dB/oct) band-pass filter (Neuroscan Edit 4.3). Data were initially visually inspected and sections containing excessive artifacts were manually rejected. Subsequently, after excluding the first five stimuli of intensity to reduce short-term habituation effects, EEG were segmented into periods of 600 ms, starting 100 ms prior to stimulus onset. The five stimuli intensities were averaged separately. Segments with amplitudes exceeding ±50 *μ*V were rejected from further analysis. ERP averages were computed for each stimulus intensity level. Mean sweep numbers for 60 dB to 100 dB ranged between 75.5 and 86.5.

We restricted our analysis to the Cz electrode, the most commonly used in LDAEP research [25]. N1 was defined as the most negative amplitude from 60 to 160 ms and P2 was defined as the most positive amplitude from 120 to 250 ms. The peak-to-peak N1/P2 amplitude was calculated as the difference in amplitude between N1 and P2 peaks. The amplitude/stimulus intensity function slopes (ASF slope) of N1, P2, and N1/P2 were calculated by linear regression, with stimulus intensity being the independent variable, and N1, P2, and N1/P2 amplitudes being the dependent variables.

### 2.5. Statistics Analysis

All statistical analyses were performed using SPSS 16.0. The amplitudes of N1, P2, and N1/P2 were analyzed in separate analyses of covariance (ANCOVA) with stimulus intensities (60, 70, 80, 90, and 100 dB) as within factor and group (high optimistic/pessimistic) as between factor, controlling for the effect of gender [26]. Reported results were restricted to effects involving the group factor and group-intensity interactions [8]. Group effects indicated differences in mean amplitudes between the optimistic and pessimistic groups while group-intensity interactions indicated differences in the ASF slopes between optimistic and pessimistic subjects. Differences of ASF slopes between high optimistic and high pessimistic groups were compared by one-way analyses of variance (ANOVA), controlling for the effect of gender. The relationships between the ASF slopes and OPS-C scores were assessed using Spearman correlations.

## 3. Results

### 3.1. Rating Scales

There were no significant differences between high optimistic and pessimistic groups with regard to gender, age, SDS, and SAS score (see Table 1).

### 3.2. ERP Evaluation

**Figure 1:**
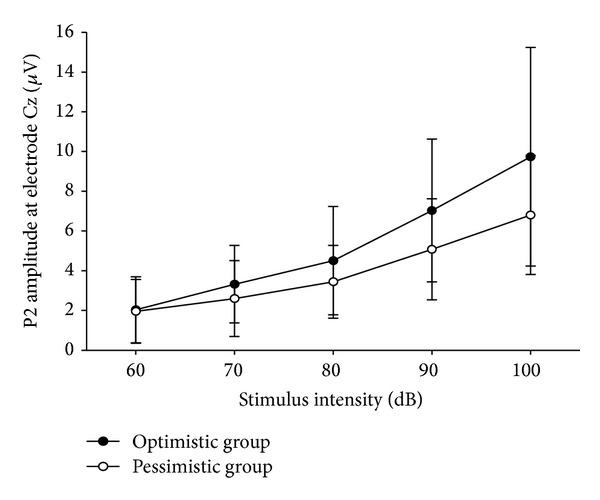
Mean amplitude over five stimulus intensities for the optimistic and pessimistic groups at electrode Cz.

**Figure 2:**
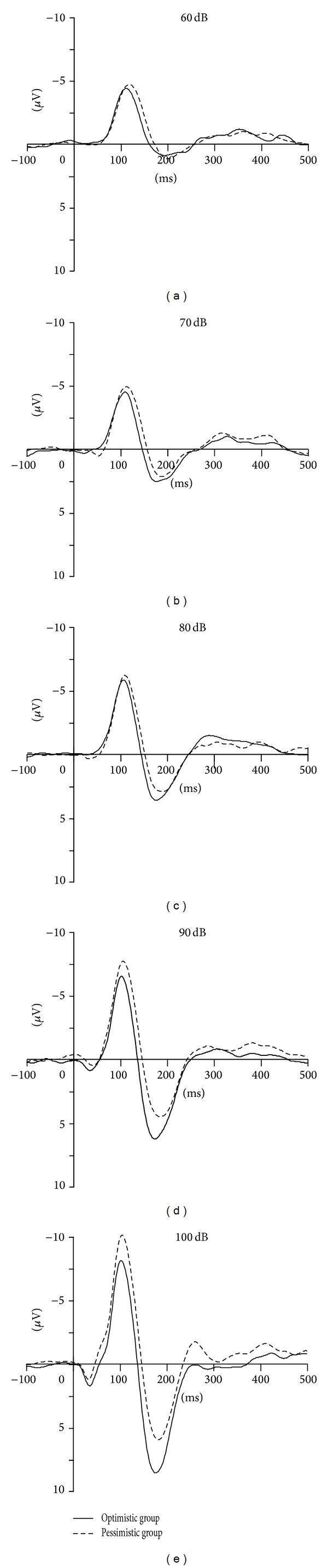
Comparison of high optimistic and high pessimistic groups P2 component averages at electrode CZ. Alongside the change of stimulus intensity (from 60 to 100 dB), the amplitude difference of the P2 component between groups increased.

Table 2 depicts the ERP data. Figures 1 and 2 showed the mean amplitudes over the five stimulus intensities for the optimistic group and pessimistic group at electrode Cz. Three analyses of variance were performed for N1, P2, and N1/P2 peaks, respectively. No significant differences were found between group factors for N1, P2, or N1/P2 amplitudes. A significant effect for group×intensity interaction was found for P2 amplitudes (corrected by Greenhouse-Geisser, *F*(1.88, 84.53) = 3.27, *P* = 0.046). However, there was not such an interaction for N1 and N1/P2 peak amplitudes. One-way ANOVA showed that P2 ASF slope was significantly higher in optimistic group than that in pessimistic group (with *F*(1,45) = 5.07, *P* = 0.029) (see Table 2 and Figure 1). No significant differences were found between groups for the N1 and N1/P2 ASF slopes (with *F*(1,45) = 2.69, *P* = 0.108 and *F*(1,45) = 1.02, *P* = 0.318, resp.)

### 3.3. Gender Effect

The OPS-C scores of participants did not vary by gender (*P* = 0.359) (Table 1). There were significant effects of gender on the P2 and N1/P2 amplitudes (*P* = 0.002 and 0.011, resp.). The gender effect was not significant on the N1 amplitude (*P* = 0.474). Besides, significant gender effects were also found on the N1 and N1/P2 ASF slopes, and the mean ASF slopes were higher for female students than male students (*P* = 0.023 and 0.002, resp.). However, gender did not exert a significant effect on P2 ASF slope (*P* = 0.074).

### 3.4. Correlations between OPS-C Scores and ASF-Slopes

When all subjects were considered as a group, the ASF-slopes of P2 were positively correlated with OPS-C scores (*r* = 0.297, *P* = 0.041). No significant correlations were found between slopes of N1 or N1/P2 and OPS-C scores. When only high optimistic or pessimistic subjects were considered, no significant correlations were found between the OPS-C scores and any ASF-slopes of N1, P2, or N1/P2.

## 4. Discussion

This is the first study to measure the ASF-slope of optimistic and pessimistic traits in college students. Our results are in agreement with previous reports [27], finding that the amplitudes of the N1, P2, and N1/P2 increased with increasing intensity. There were no significant differences in the amplitudes of the N1, P2, or N1/P2 components at any intensity level site between groups. Similar to previous results [28], we found that although N1 and N1/P2 ASF slopes did not differ significantly between groups, there was a significant difference for the P2 ASF slopes between the two groups, probably indicating a lower level of serotonergic neurotransmission in the optimistic group subjects [7]. Beauducel et al., in reporting the impact of several methodological variations used in the assessment of LDAEP, stated that, if measured reliably, P2 slopes may reflect stimulus intensity changes more precisely than N1/P2 slopes [25]. This may apply to our findings.

Physiologically, the N1 represents early orienting to new external stimuli and the P2 is related to early aspects of selective attention when processing information [7]. Increasing of the P2 amplitudes is hypothesized to indicate reduced serotonergic neurotransmission in the central nervous system [29]. Both shallow and steep LDAEP have been assumed to be a consequence of hypothetical central mechanism, which regulates the sensory sensitivity and is most likely reflected by the 5-HT system [7]. According to it, a shallow LDAEP reflects an over-activity of the central mechanism that protects the organism from sensory overload; however, a steep LDAEP reflects a lack of such protection mechanism. Therefore, the shallow LDAEP in high pessimistic college students in our results might indicate a prominent activity of this regulating mechanism, reflecting an enhanced activity of neuronal firing of the serotonergic neurons in the primary auditory cortex [29]. Similar to the interpretation to impulsivity, higher LDAEP in optimistic group might result from the lower serotonergic preactivation and losing its protection function against sensory overstimulation as stimulus intensity increases [29]. Several researches have documented that 5-HT exerts an inhibitory influence on the positive and negative effects rather than only inhabiting negative state in healthy males [30, 31]. It is possible that the pessimistic students were more prepared to enter a state of protective inhibition due to high level serotonergic neurotransmission when presented with high intensity stimuli in an uncontrollable situation [32], while students in the optimistic group may have a weak cortical inhibitory system to protect against overstimulation due to low inhibition function of 5-HT [33].

Friedman and Meares found that there was a greater intensity dependence of cortical evoked potentials in extraverts compared to introverts, supporting the current findings [15]. Brocke et al.'s study also reported that sensation seeking (the seeking of novel, varied sensations, and the willingness to take social and physical risks to pursue such experiences) was positively correlated with a steeper LDAEP [34]. A close relationship has been shown between sensation seeking and the optimistic trait [35, 36]. Besides, Meyer et al. reported that the severity of pessimism for depressive subjects was negatively correlated with the levels of 5-HT agonists in the brain cortex by positron emission tomography (PET) [37]. So, although pessimism was mainly associated with the tendency of depression [14], the correlations between optimism/pessimism and likelihood of depression in healthy individuals should be studied further in the following studies.

Possible differences in genotypes of the participants may account for part of our results. Allelic variation in the promoter region of the serotonin transporter gene (5-HTTLPR) was associated with different positive and negative affective traits [18]. LL individuals showed a marked preference to process positive affective materials and avoid negative affective materials. This protective pattern was not obvious among individuals carrying the short allele (SS or SL) [18]. Both Strobel et al. and Hensch et al. found that individuals with the LL genotype exhibited a stronger intensity dependence on the auditory evoked potential compared to individuals with the SS or SL genotype [19, 20]. We speculate that there is a higher frequency of LL genotype carriers in optimistic subjects, whereas SS or SL genotype might be overrepresented in pessimistic subjects. Recently, a meta-analysis has shown that depression was positively associated with the short allele of the 5-HTTLPR and negatively with long allele in humans [38].

The proportion of males was 41.6% in the optimistic group and 58.4% in the pessimistic group (*P* = 0.248). In our total sample (*n* = 495; 271 male and 224 female), the OPS-C scores did not vary by gender (*P* = 0.306). In our ERP study, the OPS-C scores also did not vary by gender (*P* = 0.359) (Table 1). Findings that the mean ASF slopes were higher for female students than male students for the N1 and N1/P2 ASF slopes corroborated a study of gender differences in the LDAEP by Oliva et al. [26]. The authors reported that the mean N1/P2 slopes for female participants were higher than those for male participants (*P* < 0.0001), suggesting that the LDAEP was modulated by gender associated differences in serotonin transmitters. In our study, gender did not exert a significant effect on P2 ASF slope. However, there was a significant difference on P2 ASF slope between the optimistic and pessimistic groups, which needs to be further studied in the following study.

In our results, a significant correlations were present between the P2 ASF slope and the total OPS-C scores when all the participants were taken as a whole; however, no significant correlations were found between total OPS-C scores and the N1 or N1/P2 components either for the whole sample or the separate group. Actually, we classified our healthy participants into optimistic trait and pessimistic trait by a one-dimensional rather than a two-dimensional Optimism-Pessimism Scale, which is the higher the total scores the more optimistic a person is and vice versa. We have just included a small part of subjects with extreme high (*n* = 24) and low scores (*n* = 24) of each trait. The OPS-C scores in optimistic group mainly lay between 100 and 104 and those in pessimistic group mainly lay between 86 and 90. The full ranges of OPS-C scores in both groups were much narrow and might not be able to obtain a significant or large correlation with the ASF-slopes. Thus, from the view of general human beings, our results, to some extent, might be indicating a trend that the higher the scores of a person, the higher the ASF-slope of P2.

There are some limitations of this study. Firstly, the sample was small, consisting of only 48 college students; there was a relatively low power to detect differences and therefore; the validity of the research may be limited in comparison to larger studies. Secondly, the effects of the activity of the noradrenergic and dopaminergic systems were not taken into consideration. The literature has demonstrated that these systems might associate with individuals' LDAEP [39] and a report has implicated that dopamine might enhance a person's optimism bias [40].

## 5. Conclusions

Our results suggest that the serotonergic neurotransmission of the high optimistic college students was inferior to that of the pessimistic ones. Further investigations are needed to provide sufficient supports for our results.

## Figures and Tables

**Table 1:** Characteristics of the optimism and pessimism group.

	Optimism (*n* = 24) (M ± SD)	Pessimism (*n* = 24) (M ± SD)	*χ* ^2^	*t* value	*P* value
Gender (m/f)	10/14	14/10	1.333		0.248
Age (y)	19.79 ± 0.93	20.08 ± 0.93		−1.086	0.283
OPS-C	102.63 ± 3.63	87.67 ± 2.90		15.765	*P* < 0.001
SDS	32.79 ± 4.78	33.90 ± 5.13		−0.750	0.616
SAS	30.67 ± 3.37	31.24 ± 5.37		−0.421	0.738

OPS-C: the Chinese version of the Optimism-Pessimism Scale; SDS: the Self-rating Depression Scale and; SAS: the Self-rating Anxiety Scale; M and SD stand for mean and standard deviation; *P* = 0.05.

**Table 2:** ERP Data: N1, P2 amplitudes and N1, P2, N1/P2 amplitude slopes.

	Optimistic group (*n* = 24)	Pessimistic group (*n* = 24)
	(Mean ± SD)	(Mean ± SD)
Amplitude 60 dB/100 dB/SPL (*μ*V)^1^		
N1	4.97 ± 2.09/9.01 ± 3.30	5.11 ± 2.10/10.00 ± 3.74
P2	2.03 ± 1.66/9.73 ± 5.50	1.96 ± 1.61/6.80 ± 2.99

Amplitude slope (*μ*V/10 dB)^2^		
N1	1.02 ± 0.72	1.26 ± 0.64
P2	1.91 ± 1.20	1.21 ± 0.61
N1*/*P2	2.93 ± 1.18	2.47 ± 1.03

Note: ^1^The lowest (60 dB) and the highest (100 dB) stimulus intensities of N1 and P2 amplitudes data are given at Cz in *μ*V.

^2^Values of the mean amplitude slopes increases were given within the five stimulus intensities in *μ*V per 10 dB.
